# Transition to adulthood with a bladder augmentation: histopathologic concerns

**DOI:** 10.1590/S1677-5538.IBJU.2016.0548

**Published:** 2017

**Authors:** Emil Mammadov, Sergulen Dervisoglu, Mehmet Elicevik, Haluk Emir, Yunus Soylet, S. N. Cenk Buyukunal

**Affiliations:** 1 Department of Pediatric Surgery, Near East University Medical Faculty, Turkey; 2 Department of Pathology, Cerrahpasa Medical Faculty, Istanbul University, Istanbul, Turkey; 3 Department of Pediatric Surgery, Division of Pediatric Urology, Cerrahpasa Medical Faculty, Istanbul University, Istanbul, Turkey

**Keywords:** Urinary Bladder, Neoplasms, Pediatrics

## Abstract

**Aim:**

To investigate the histopathologic changes in native bladder and gastrointestinal segment, the relation between histopathologic changes, type of operation and the period passed over operation in patients with bladder augmentation.

**Materials and methods:**

Twenty consecutive patients were enrolled in this study. Histopathologic evaluation of the cystoscopic mucosal biopsies from native bladder and enteric augment was performed in all patients.

**Results:**

Active or chronic non-specific inflammation of various degrees was found in all specimens except two. Metaplastic changes were detected in 3 patients. Two patients had squamous metaplasia (one focal, one extensive) and one patient had intestinal metaplasia. All metaplastic changes were found in native bladder specimens. The type of augmentation in patients with metaplastic changes were ileocystoplasty and sigmoidocystoplasty. No signs of malignancy were detected in any patient.

**Conclusion:**

The complexity of the disorders requiring bladder augmentation does not let the surgeons to draw a clear line between different groups of complications including malignancy formation. However, due to challenging course of the augmentation procedure itself, surgeons should be well aware of the possibility of malignancy development.

## INTRODUCTION

Bladder augmentation gained a widespread acceptance especially after implementation of clean intermittent catheterization procedure by Lapides in 1972 (1). This simple procedure was a revolutionary step in medicine and reasonably increased the popularity of bladder augmentations.

The main indications for bladder augmentation include decreased bladder capacity and compliance, increased intravesical pressure and detrusor overactivity. The operation itself is complex and requires high surgical skills. Early and late postoperative courses are not innocent either. Each phase after surgery has a different set of complications like perforation, mucus and stone formation, metabolic disorders, urinary incontinence, hematuria-dysuria, gastrointestinal system related complications, growth alteration and tumor formation.

Traditional belief that augmented bladder possesses the risk for tumor formation is still debated by scientific authorities. Due to complexity of the diseases requiring bladder augmentation it is hard to tell whether tumor formation in neobladder is directly related to the procedure itself. Several studies with various results have been conducted by different authors (2-9).

The aim of this study was to investigate the histopathologic changes, the relation of histopathologic changes with type of operation and the period passed over operation in augmented bladders during adolescence and early adulthood.

## MATERIALS AND METHODS

Institutional Review Board approval was obtained prior to the study. The targeted population involved all patients who have undergone bladder augmentation in our clinic in the period between 1987-2009. The patients on routine follow up were called by phone and asked to be enrolled in the study. Informed consent was obtained and standard preoperative blood tests including renal function tests were performed in all patients. History of chronic bacteriuria, recurrent urinary tract infections and calculi were questioned prior to the procedure.

**Figure 1:**
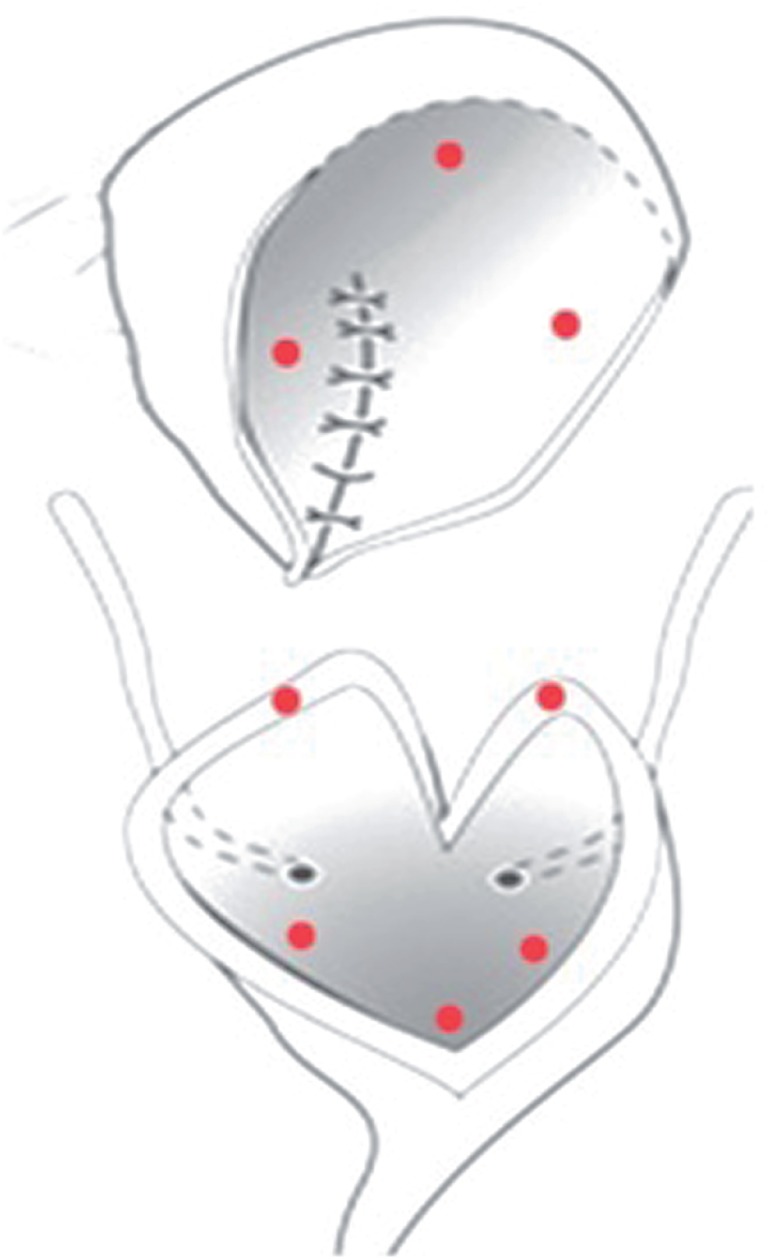
Planned sites for biopsies (triangular manner).

All cystoscopic procedures were performed under general anesthesia. The neobladder was evaluated thoroughly for calculi and the mucosa was examined for suspicious regions or lesions. Mucosal biopsies were planned to be obtained from 8 points (3 from native bladder, 3 from augmented tissue and 2 from anastomotic border) (Figure-1). The biopsies from native bladder and augmented segment were planned to be taken from 3 points each forming a virtual triangle. This was done to address the maximum possible area in each segment. Visual emphasis was made on the anastomotic border.

The specimens were sent to the Pathology Department where standard staining procedures (hematoxylin-eosin) were applied. The biopsy samples were examined for inflammation, fibrosis, metaplasia dysplasia and signs of malignancy.

## RESULTS

Seventy-seven patients have undergone bladder augmentation in our clinic in the period between 1987-2009. Patients who were lost during follow-up, patients who could not be reached and who rejected to participate were excluded, leaving 20 patients who were considered eligible for the study.

The study group consisted of 13 male and 7 female patients with the mean age of 20 (range: 11-29) who have undergone bladder augmentation for bladder exstrophy (8) and neurogenic bladder (10). Two other patients with long gap urethral injuries and severe bladder neck ruptures due to pedestrian type traffic accident also had neurogenic type bladder problems which were related with the severity of trauma or the side effects of correctional surgical procedures. The distribution of the patients according to the interval passed over the augmentation was as follows:

1. Less than 5 years after the augmentation: 4 patients (4 ileal augmentations);
2. 5-10 years after the augmentation: 5 patients (2 ileal, 1 ileogastric, 1 gastric, 1 sigmoid augmentations);
3. More than 10 years after the augmentation: 11 patients (8 ileal, 1 ileogastric, 2 sigmoid augmentations).

Preoperative complete blood count and coagulation panel results were normal in all patients. Two patients had previously diagnosed chronic renal failure at the time of evaluation. Three patients had bladder stones and 4 patients had chronic asymptomatic bacteriuria. Twelve patients have experienced recurrent urinary tract infections either prior to or after the augmentation procedure.

The biopsies were obtained by cystoscopy in 17 patients and through open approach during planned surgery in 3 patients (2 bladder stone extractions and 1 bladder neck disconnection). The rigid cystoscope was introduced either via urethra (1 patient) or via Mitrofanoff stoma (16 patients) in patients with reconstructed or disconnected bladder neck. No complications associated with the biopsy procedure were noted postoperatively.

There weren't any suspicious macroscopic lesions in any patient. The intestinal mucosa is usually easily distinguished from native bladder by its color and villous appearance. However, it was impossible to differentiate native bladder mucosa from intestinal mucosa visually during cystoscopy in 6 patients. The biopsies were taken from the area closest to bladder neck in those patients presuming that it would be the native bladder mucosa but all those biopsies came out to be belonging to augmented intestinal tissue. Additionally, 3 patient's biopsies taken from “visually confirmed” native bladder mucosa revealed augmented intestinal tissue (Table-1).

**Table-1:** Histopathologic findings (Table separated according to years passed over augmentation).

Age-Gender	Period over augment (years)	Segment	Macroscopy	Native Bladder	Augment
28-♂	1	Ileal	No significant feature	Intestinal tissue*	Chronic inflammation
21-♂	4	Ileal	No significant feature	Intestinal tissue*	Chronic inflammation, fibrosis
15-♀	4	Ileal	No significant feature	Active inflammation	Active inflammation
23- ♀	4	Ileal	No significant feature	Extensive squamous metaplasia	Active inflammation
21-♀	6	Ileal	No significant feature	Intestinal tissue*	Active inflammation
19-♀	6	Ileogastric	No significant feature	Intestinal tissue*	Chronic inflammation
11-♂	8	Ileal	No significant feature	Chronic inflammation	Chronic inflammation
22-♂	8	Gastric	No significant feature	Intestinal tissue*	Active inflammation
19-♀	9	Sigmoid	No significant feature	Intestinal tissue*	No inflammation
20-♂	10	Ileal	No significant feature	Active inflammation	Active inflammation
26-♂	10	Sigmoid	No significant feature	Chronic inflammation	Chronic inflammation
12-♂	11	Ileal	No significant feature	No inflammation	Active inflammation
29-♂	11	Ileal	No significant feature	Focal intestinal metaplasia	Active inflammation
17-♂	11	Ileal	No significant feature	Intestinal tissue*	Active inflammation
23-♀	12	Ileal	No significant feature	Intestinal tissue*	Active inflammation
17-♂	12	Ileal	No significant feature	No inflammation, fibrosis	Chronic inflammation
29-♀	13	Ileogastric	No significant feature	Intestinal tissue*	Active inflammation
22-♂	14	Ileal	No significant feature	Active inflammation	Active inflammation
16-♂	15	Ileal	No significant feature	Active inflammation	Active inflammation
25-♂	23	Sigmoid	No significant feature	Focal squamous metaplasia	Active inflammation

* biopsies with presumed native bladder tissue coming out as intestinal tissue (possible intestinalization)

**Figure 2:**
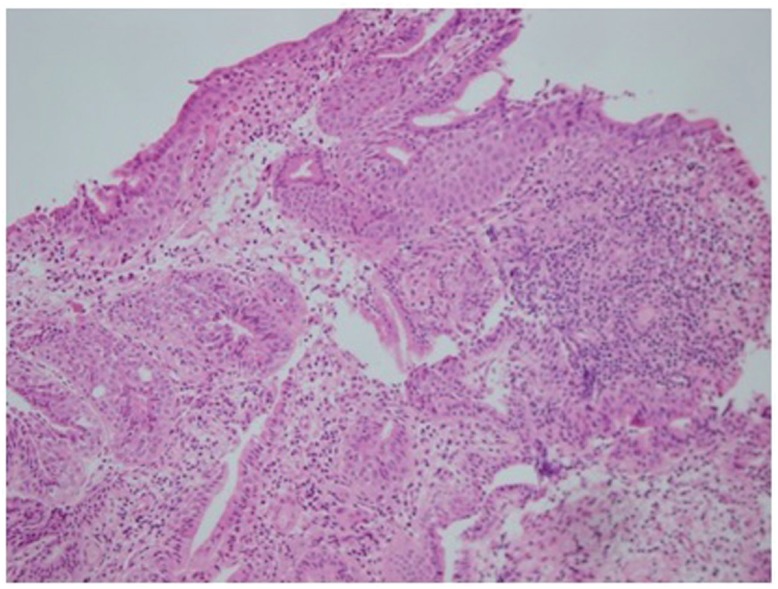
Focal intestinal metaplasia (H-E staining, 200 magnification).

The histopathology was evaluated using hematoxylin-eosin staining. All specimens except two showed various degrees of active or chronic non-specific inflammation. Different types of metaplastic changes were detected in 3 patients. All three patients were in exstrophy-epispadias complex group and had staged repair prior to augmentation. All three patients had Mitrofanoff procedure during the augmentation. Additionally, two of them have undergone multiple operations after the augmentation (bladder neck procedures, post epispadias fistula repair etc.). Two patients had squamous metaplasia (one focal, one extensive) and one patient had intestinal metaplasia. All three metaplastic changes were found in the native bladder. Squamous metaplasia was detected in patients with ileocystoplasty and sigmoidocystoplasty. Interestingly the patient with extensive squamous metaplasia had undergone ileocystoplasty just four years prior to current evaluation. The patient with intestinal metaplasia had ileocystoplasty as procedure of choice (Figure-2).

The patient with previous sigmoidocystoplasty who had squamous metaplasia formation, had an additional stone formation as well (2×2.5×2cm). Pediatric Pathology Division commented that those histopathologic changes could be related with irritation process which was created by the stone. After the removal of stone patient had no clinical problem. The parents did not accept another biopsy examination after the stone removal, since they did not want another intervention under general anesthesia. Ultrasound exams and routine urine analyses and cultures revealed no major problem in this patient. In other two patients, which were treated for stone formation, histopathologic exam of the biopsy specimens revealed only chronic non-specific inflammatory changes as in the other 18 patients. For these two patients, it was decided to perform the routine follow-up with urine analysis, cultures and ultrasonographic exams. No additional problem was detected in these 2 patients.

No neoplastic changes or active tumor formation were detected in any patient (Table-1).

## DISCUSSION

Malignancy development incidence in the published series ranges from 1.2 to 4.5%. Several hypotheses were proposed to explain tumor development. Inflammation secondary to chronic bacteriuria yielding cancerogenic nitrosamines, chronic contact of urine with the intestinal epithelium resulting in chronic irritation and interaction between two different types of epithelia leading to changes in intercellular interactions may contribute to malignant degeneration. Of course, many other factors like irritation from chronic catheterization, chronic asymptomatic bladder stones and mucus impaction may lead to development of chronic changes in the epithelial lining of both surfaces (2-9).

Several individual case reports and reviews on tumor development after bladder augmentation raised the concern on this issue (10-18). There are not many series in the literature regarding malignancy development following bladder augmentation. Thus, the series that have been published before were of extreme importance to us (Table-2).

**Table-2:** Published routine cystoscopic surveillance series.

Author	Number of patients	Number of malignancies	Types of malignancies
Soergel et al. (3)	260	3	Transitional cell carcinoma
Husmann et al. (4)	153	7	Transitional cell carcinoma, enteric adenocarcinoma
Higuchi et al. (5)	65	None	-
Kispal et al. (6)	54	1	Enteric adenocarcinoma

Routine surveillance in patients with bladder augmentation is still a debate among scientific authorities. From the published literature, it seems that malignancies in patients with augmentation cystoplasty have aggressive progression and have to be diagnosed early. At the same time, some authors claim that malignancy rates are low and routine surveillance is a burden for both patient and government.

Soergel et al. in 2004 published a study where the authors detected 3 transitional cell carcinomas among 260 patients with bladder augmentations (3). The tumor development could not be associated to other predisposing factors. The authors in the study proposed that bladder augmentation alone could be the cause of neoplastic development.

In 2008 Husmann et al. published another series where the authors detected 7 malignant tumors in 153 patients with bladder augmentation. The augmented enteric segment born adenocarcinomas were predominant in that study. There were 5 adenocarcinomas and 2 transitional cell carcinomas in total. In this study, direct correlation between augmentation and malignancy could not be proved due to various other predisposing factors like tobacco use and long term immunosuppression. The other probable predisposing factor stated in that study was bladder exstrophy (4).

Higuchi et al. proposed that annual surveillance of bladder tumors after enteroplasty is not cost effective due to low incidence of tumor formation. In a well-constructed long term study including 250 surveillance endoscopic evaluations, only 4 (1.6%) lesions were identified and none of them came out to be malignant (5).

More recently, Kispal et al. surveilled 54 patients with augmentation cystoplasty and detected metaplasia in 3, dysplasia in 6 and colonic segment adenocarcinoma in one patient. Metaplasia was seen in native bladder, enteric patch and anastomotic line. Dysplasia was detected mainly in the anastomotic region. The malignancy was detected 11 years after the augmentation procedure (6).

The difference in malignancy rates, study designs and low number of cases does not let the proposal of a consensus on malignancy surveillance of the patients with augmentation cystoplasty. Staying on the safe side urges pediatric urologists to perform routine cystoscopic evaluation especially when more than 10 years are passed over augmentation. The need for cystoscopic surveillance is not a debate itself, but planning of its frequency is.

The main emphasis of our study was made on histopathologic changes in neobladder in patients who were in the age group with no apparent risk for malignancy development. Our early and late complication rates in 77 bladder augmentation patients, regarding urinary incontinence, urolithiasis, metabolic issues, hematuria dysuria syndrome, bladder perforations, Mitrofanoff stoma problems, capacity and compliance problems have been reported previously (19).

In our study, we did not detect malignancy in any of the 20 consecutive patients. However, we have shown different metaplastic changes in 3 patients. Two patients had squamous metaplasia and one had intestinal metaplasia. Intestinal metaplasia in bladder has always been a debate regarding whether it is precancerous or not. Some authors state that intestinal metaplasia is a precancerous change leading to adenocarcinoma. In contrary, Corica et al. published a series of 10 years-follow-up of 53 patients with intestinal metaplasia with zero malignancy development rate (20). Still we try to stay at the safe site of this debate and approach the patients with metaplasia with extra attention.

Two patients in our series with squamous metaplasia in native bladder raised a different concern in our minds. Both patients were bladder exstrophy patients and have undergone several operations including bladder exstrophy and epispadias repair and bladder neck reconstruction procedures. One patient had several interventions for bladder stones at different times of disease course and this patient also developed vesicocutaneous fistula and was operated several times for that pathology. Even the problematic disease course of those two patients may have been the reason for metaplastic changes.

Regarding stone formation, all 3 stone patients developed during teenage period. In this age group, we noted a resistance against routine catheterizations and irrigations which we advise routinely for clearance of mucus. Thus, most of the stone formations and problems related with mucus accumulationswere mainly seen in these young patients. This problem usually disappeared when they became 16-17 years of age.

The type of augmentation in patients with metaplastic changes was ileocystoplasty and sigmoidocystoplasty. There was no obvious relation with the type of intestinal patch and underlying problem (like exstrophy or neurogenic bladder) as far as histopathologic changes were concerned. Also, literature data is not enough to conclude if the type of augmented tissue has an impact on metaplastic changes or malignancy formation.

The other interesting observation during this study was the inability to detect native bladder mucosa histologically or visualize it during cystoscopy. This fact was observed in 9 of 20 patients. One possible mechanism for that issue might be a very small bladder (as in bladder exstrophies) during the augmentation or multiple bladder neck operations which eventually reduced the size of native bladder. The other, yet unproven hypothesis which came to our mind during the study was the “mucosal changes in bladder mucosa to enteric mucosa” or “intestinalization of neobladder”. This hypothesis only raised a scientific interest during the study but the proof of this issue seemed impossible because a full thickness biopsy would be needed to prove that. Yet this hypothesis may be a beginning for a design of in vitro cell and tissue culture study.

There were several limitations of our study. The most important was the number of the patients that could be enrolled in the study. The uneven distribution of patients in each group regarding period passed over augmentation made it impossible to form a statistically healthy cohort. The other important limitation of the study was the rigid cystoscope that we had to use due to unavailability of the flexible cystoscope. To solve this issue, we planned the biopsy sites in triangular manner both in native bladder and enteric augment to address the widest area. However, inability to visualize periphery of the Mitrofanoff insertion point stayed as a drawback that we couldn't solve.

Chronic inflammatory changes were seen almost in all our patients even in late period. Stone formation may be an initiating factor, as far as metaplastic changes are concerned. However, we did not find an obvious relation between those two issues and at the same time there was no obvious relation between the intestinal or colonic patch which was used for augmentation and the type of inflammatory and metaplastic changes. Also, the Mitrofanoff procedure doesn't seem to have impact on secondary histopathological changes. However, exstrophy-epispadias complex may be a predisposing risk factor for metaplastic changes and potential malignancy. Still, we need more data to prove this hypothesis.

This is the first long-term histologic evaluation study of bladder augmentation in Turkey. In past decade, all patients in our clinic whose bladder augmentations were performed even less than 10 years ago were followed on a standard protocol which included annual radiologic screening and cystoscopic evaluation. However, during the progress of the study and with the new scientific data from the literature the annual cystoscopic evaluation in patients with less than 5 years after the augmentation was reserved to the patients with clinical complaints.

## CONCLUSIONS

We think that malignant transformation in native or augmented patch of the neobladder is a serious long-term complication. Our initial strategy was to investigate those patients with cystoscopic examinations in late postoperative period. However, due to recent publications and our experience, we changed our approach (5, 6). Our recent protocol is to perform cystoscopy only in symptomatic patients (e.g. hematuria, dysuria and any suspicious lesion in imaging studies). This kind of follow-up seems to be more comfortable to the patients and less costly to the government. At the same time, a better follow-up education program for adolescent patients aiming to solve the problem of “resistance against catheterization and irrigation” will decrease the mucus and stone formation problems. For that reason, a careful individualized follow-up of this group should be the strategy of choice for the next couple of decades.
